# An ultrasonic nanobubble-mediated PNP/fludarabine suicide gene system: A new approach for the treatment of hepatocellular carcinoma

**DOI:** 10.1371/journal.pone.0196686

**Published:** 2018-05-02

**Authors:** Bo Zhang, Mingna Chen, Youming Zhang, Wei Chen, Lihua Zhang, Lv Chen

**Affiliations:** 1 Department of Ultrasonic Imaging, Xiangya Hospital, Central South University, Changsha, Hunan, China; 2 Department of Radiology, Xiangya Hospital, Central South University, Changsha, Hunan, China; 3 Hepatobiliary and Enteric Surgery Research Center, Xiangya Hospital, Central South University, Changsha, China; 4 Department of Occupational and Environmental Health, School of Public Health, Central South University, Changsha, Hunan, PR China; National University Singapore Yong Loo Lin School of Medicine, SINGAPORE

## Abstract

**Objective:**

The purpose of this study is to generate an ultrasonic nanobubble (NB)-mediated purine nucleoside phosphorylase (PNP)/fludarabine suicide gene system for the treatment of human hepatocellular carcinoma (HCC).

**Methods:**

NBs were prepared from a mixture the phospholipids 1,2-dipalmitoyl-sn-glycero-3-phosphocholine (DPPC) and 1,2-dipalmitoyl-sn-glycero-3-phosphate (DPPA), perfluoropropane gas and other materials using the high shear dispersion method. NBs treated with ultrasound irradiation functioned as a gene-transfer system, and a self-constructed suicide gene expression plasmid, pcDNA3.1(+)/PNP, treated with fludarabine functioned as a therapeutic gene. This system was used to determine the cytotoxic effects of PNP/fludarabine on HepG2 cells and SMMC7721 cells.

**Results:**

**1.** NBs with a small diameter (208–416 nm) and at a high concentration and fine homogeneity were prepared under the optimal method. **2.** The pcDNA3.1(+)/PNP plasmid was efficiently transfected into HCC cells using ultrasonic NBs. **3.** At 0.75μg/ml fludarabine, PNP/fludarabine showed marked cytotoxic effects toward HepG2 and SMMC7721 cells. PNP/fludarabine achieved the same effect against both SMMC7721 and HepG2 cells but at a lower concentration of fludarabine for the latter. **4.** Bystander effects: a 10–20% decrease in the cell survival rate was observed when only 5–10% of transfected cells were PNP positive.

**Conclusions:**

NBs constitute a non-toxic, stable and effective gene-delivery platform. The PNP/fludarabine suicide gene system inhibited the growth of HCC cells, induced HCC cell apoptosis, and caused a notable bystander effect at a low fludarabine concentration. This study establishes an important new method for miniaturizing microbubbles and improving a new NB-mediated approach for gene therapy of HCC.

## Introduction

Hepatocellular carcinoma (HCC) is the fifth most common malignancy and the third leading cause of cancer-related death[1]. More than 700,000 new cases are diagnosed each year worldwide, and unfortunately, more than 600,000 deaths annually are attributed to HCC[2]. The current predisposing conditions and major risk factors are clearly defined as hepatitis C virus (HCV) and hepatitis B virus (HBV) infections[3, 4]. Although curative treatments, such as liver transplantation, surgical resection or ablation, have achieved great progress, the recurrence, metastasis, and mortality of HCC remain high. Thus, gene therapy using “suicide” genes is increasingly being considered a feasible proposal because of its apoptosis-related mechanisms and “bystander effect”[5]. However, as gene therapy has been clinically limited by non-targeted and insufficient gene transfer, it is important to develop a method for the precise monitoring of therapeutic gene expression. One such approach is ultrasound-targeted microbubble destruction, a non-invasive, efficient, targeted and safe transfer technique that delivers plasmids to specific tissues[6, 7]. Microbubbles burst in the presence of ultrasound irradiation, allowing the target gene to be released and enter tumor cells. Tumor vessels lack tight junctions, and the diameter of these vessels ranges from 380 to 780 nm[8]. However, microbubbles (MBs, such as SonoVue) range from 1 to 10 μm in diameter, and nanoscale particles range in size from 10 to 1000 nm. Thus, NBs can potentially extravasate through the capillary barrier to reach cells at the tumor site for targeted drug delivery.

Purine nucleoside phosphorylase (PNP) converts adenosine analogs into highly cytotoxic metabolites, which are then incorporated into both DNA and RNA, inhibiting DNA, RNA and protein synthesis and ultimately inducing apoptosis[9, 10]. PNP converts the purine ribonucleoside prodrug fludarabine phosphate into the highly toxic agent 2-fluoroadenine, a molecule that freely diffuses across cell membranes, allowing it to spread from PNP-transduced to untransduced cells. Moreover, this compound is toxic to both proliferating and non-proliferating cells[10], thereby achieving a potent “bystander effect”. Compared to other suicide gene systems, PNP/fludarabine has more powerful tumor lethality and security[11].

Few studies to date have evaluated the therapeutic potential of *Escherichia coli *PNP/fludarabine as a suicide gene to destroy HCC cells. In particular, the use of an ultrasonic NB-mediated PNP/fludarabine suicide gene system as a potential efficient and secure strategy for gene therapy of HCC has never been reported internationally. In this study, we explore a method for producing ultrasonic NBs and the feasibility of using the ultrasonic NB-mediated PNP/fludarabine suicide gene system for HCC gene therapy in vitro, a technique that has not yet been reported.

## Materials and methods

### Nanoscale perfluorinated propane lipid microbubble preparation

Different concentrations of 1,2-dipalmitoyl-sn-glycero-3-phosphocholine (DPPC; Avanti Polar Lipids Inc., Alabaster, Alabama, USA), Tween 80 (T_80_, Beibei Chemical Factory, Chongqing, China) and Span 60 (S_60_, Beibei Chemical Factory, Chongqing, China) were added to a mortar (T_80_/S_60_ in 24:8, 20:10, 15:15), and 15 ml of phosphate-buffered saline (PBS; pH 7.4) was added. The mixture was ground into an emulsion, and PBS was added to 250 ml. The mixture was then dispersed and emulsified at 45,000 rpm using an ultrasonic dispersion instrument (MEICO Fisher Scientific, Massachusetts,USA) and sealed with nitrogen (purity>99.99%, Guangzhou gas plant, Guangzhou, China) after 2 min. The liquid was injected with perfluorinated propane gas (purity>99.99%, Tianjin pan-fluorine international trade co. LTD, Tianjin, China) for approximately 2 min at rate of 1 ml/sec. The above emulsion was dispersed at different shear rates and times using a tool (F22A high-shear dispersion and emulsifying homogenizer, FLUKE Corp., Germany) placed in the liquid (Table 1). Finally, the substratum white emulsion was stored in a centrifuge tube at 0–4°C.

**Table 1:** Uniform design experiment for ultrasonic NBs.

Group	High shear time (min)	High shear rate (rpm)	Concentration of DPPC (mg/50 ml)	T_80_/S_60_
1	3	14000	20	3
2	3	22000	25	2
3	3	22000	15	1
4	5	30000	25	1
5	5	14000	15	3
6	5	14000	20	2
7	8	22000	15	2
8	8	30000	20	1
9	8	30000	25	3

Several dilutions of perfluorinated propane lipid ultrasound microbubbles were analyzed using an inversed fluorescent microscope (TE2000, Leica, Wetzlar, Germany) and a laser particle sizer (MasterSizer 2000, Malvern Instruments Ltd., Malvern, UK) to determine the particle sizes and distribution. The conditions used for the preparation were analyzed by uniform design and multiple regression analyses (Uniform Design 3.0, China) by gradually introducing methods, and the independent samples t test or variance analysis was employed to compare data between groups. Finally, the regression equation was calculated, and the optimized production process was selected.

### Cell culture and the effects of different concentrations of NBs on cell growth

The HepG2 and the SMMC 7721 human HCC cell lines were stored in our laboratory. HepG2 cells were cultured in RPMI 1640 (Gibco, Carlsbad, CA, USA) supplemented with 10% fetal calf serum (Gibco, Carlsbad, CA, USA); SMMC 7721 cells were cultured in RPMI 1640 supplemented with 10% newborn calf serum and 0.2 M glutamine. All cells were grown to logarithmic phase and maintained at 37°C in a humidified atmosphere containing 5% CO_2_.

HepG2 cells in the logarithmic growth phase were seeded in 6-well plates at a seeding density of 1×10^3^ cells/well for 24h. Each of the six wells was designated as an experimental group treated with 0.0%, 1.0%, 2.0%, 5.0%, 10.0%, and 15.0% NBs. The control group was only treated with culture medium without cells. The optical densities (ODs) of the samples in each well were measured at 570 nm using an enzyme-linked immunosorbent assay (ELISA) plate reader (6800, Bio-Rad, Hercules, California, USA).

### Green fluorescent protein (GFP) plasmid transfection using NBs or liposomes

NB-mediated GFP expression was compared with liposome-mediated GFP expression to demonstrate the effectiveness of NB transfection. HepG2 cells in the logarithmic growth phase were seeded in 6-well plates at a seeding density of 5×10^4^ cells/well for 24h as described above. The pshuttle0IRES-hrGFP-1 plasmid carrying a kanamycin resistance cassette and the GFP coding sequence was previously prepared by our laboratory and applied to detect transfection efficiency. We established five groups to compare differences in the transfection efficiency of the GFP plasmid between NBs and liposomes, as follows: 1) pure plasmid (8 μg); 2) NBs and 4 μg of plasmid; 3) NBs and 8 μg of plasmid; 4) liposomes and 4 μg of plasmid; 5) liposomes and 8 μg of plasmid. Fluorescence microscopy (counting positive cells), flow cytometry (FCM, emission wavelength of 488 nm and receiving wavelength of 530 nm; Cellquest soft was used to gain and analyze data), and quantitative real-time polymerase chain reaction (RT-qPCR: 40 cycles of denaturation at 94°C for 30 s, annealing at 62°C for 60 s, and extension at 72°C for 10 min) were employed to detect GFP expression between the two methods. All sections were independently evaluated by two investigators using a LEICA DM5000B microscope (Leica, Solms, Germany) at ×200 magnification. To minimize variability, the average of 12 high-power non-overlapping fields in each section was used for analysis. The sequences of the GFP primers were forward primer, 5'-CAACCGCACCTTCACCAAGT-3' and reverse primer, 5'-GAACATCTCCTCGATCAGGTTGA-3' (Omiga 2.0). The amplified GFP band was 151 bp.

### Transfection of the GFP plasmid with or without ultrasound irradiation

HepG2 cells in the logarithmic growth phase were seeded in 6-well plate(5×10^4^ cells/well) for 24h. We also established the following six groups to compare the transfection efficiency of the GFP plasmid in the presence or absence of ultrasound irradiation: a. pure plasmid (8μg); b. 8 μg of plasmid and ultrasound irradiation; c. NBs and 8 μg of plasmid; d NBs, 8 μg of plasmid and ultrasoundirradiation; e. liposomes and 8 μg of plasmid; f. liposomes, 8 μg of plasmid and ultrasound irradiation. We used fluorescence microscopy and FCM to detect GFP expression, and we compared the effects of ultrasound irradiation. The ultrasound parameters used were as follows: central frequency, 1.3 MHz; average intensity per cross-section, 0.5 W/cm^2^[12]; continuous ultrasound at an 0.5 W/cm^2^ output intensity for 60 s (focus on 4 cm; the distance between the ultrasonic probe and the bottom of the plate was 4 cm).

### pcDNA3.1(+)/PNP plasmid constructs

For a marked anti-tumor effect, we selected PNP as the suicide gene and the *E*. *coli* (DH5α) pcDNA3.1(+) plasmid (5428/5427 bp, ampicillin resistant cassette, provided by our laboratory) as the eukaryotic gene expression system. The sequences of the oligonucleotides primers were forward primer, 5'-CGCGGATCCATGGCTACCCCACACATT-3' and reverse primer, 5'-CCGCTCGAGTTACTCTTTATCGCCCACC-3'. An amplified PNP 748-bp fragment was digested with BamH I and Xho I and purified, and the fragment was then inserted into the pcDNA3.1(+) vector at these sites. The pcDNA3.1(+)/PNP expression plasmid was amplified, extracted, purified, and digested for confirmation.

### pcDNA3.1(+)/PNP transfection of HCC cells using ultrasonic NBs

pcDNA3.1(+)/PNP (experimental group) or pcDNA3.1(+) (control group) was transfected into overnight-cultured HCC cells using ultrasonic NBs. A control group without pcDNA3.1(+) transfection was also established. The above three groups (three wells per group, 5×10^4^ cells/well) were irradiated with 0.5 W/cm^2^-output intensity ultrasound at a frequency of 1.3 MHz for 60 s (focus of 4 cm; the distance between the ultrasonic probe and the bottom of the plate was 4 cm) at 37°C. The cells were screened by incubation with G418 (1200 μg/ml, Invitrogen) after 24 h of culture. All cells in the control group were dead after the above treatment, whereas the pcDNA3.1(+)/PNP- and pcDNA3.1(+)-transfected cells continued to proliferate. PNP expression in HCC cells was detected by RT-qPCR.

### Use of the PNP/fludarabine suicide gene system for HCC therapy in vitro

HepG2-pcDNA3.1 cells, HepG2-pcDNA3.1/PNP cells, SMMC7721-pcDNA3.1 cells, and SMMC7721-pcDNA3.1/PNP cells were selected by addition of G418 (800 μg/ml) to the culture. Six-well plates(3×10^3^ cells/well) were divided into experimental groups treated with 0, 0.05, 0.1, 0.25, 0.5, 0.75, 1, 1.25, 2.5, 5, and 10 μg/ml fludarabine. The ability of the PNP/fludarabine suicide gene system to inhibit the growth of HCC cells was detected using the 3-(4,5-dimethylthiazol-2-y1)-2,5-diphenytetrazolium bromide (MTT) assay (Sigma, Santa Clara, California, USA) with 5 mg/ml MTT. The ODs of samples in each well were measured at 570 nm to determine the cell survival rate.

In addition, the apoptosis induction of HCC cells was detected by FCM (BD Biosciences, Franklin L, New Jersey, USA).HepG2-pcDNA3.1/PNP and SMMC7721-pcDNA3.1/PNP cells in six-well plates were cultured overnight, after which the culture medium was replaced with 0.75 μg/ml fludarabine and 10% fetal calf serum. Apoptotic cells were then detected using Annexin V-FITC Apoptosis Detection Kit. The results were analyzed using Cell Quest software.

The bystander effect of the PNP/fludarabine suicide gene system to inhibit the growth of HCC cells was detected also using MTT assay. Transfected HCC cells were mixed with un-transfected HCC cells at ratios of 0/100, 2.5/97.5, 5/95, 10/90, 20/80, 30/70, 40/60 and 50/50. After 24 h, 0.75 μg/ml fludarabine was added to each well(3×10^3^ cells/well). After 5 days, the cells were subjected to the MTT colorimetric assay, and the ODs of the samples in each well were measured at 570 nm to calculate the percentage of growth inhibition.

### Statistical analysis

Data were statistically analyzed using SPSS 11.5 for Windows (SPSS, Chicago, IL, USA), and the results are presented as means±standard errors (SEM). Student’s t-test was performed to assess differences between two independent samples. Trend Chi-square test was performed to assess variable tendency of different ratios of transfected and un-transfected cells survival rate. A *P* value ≤0.05 was considered statistically significant.

## Results

### Preparation conditions and particle size distribution of NBs

**Fig 1:**
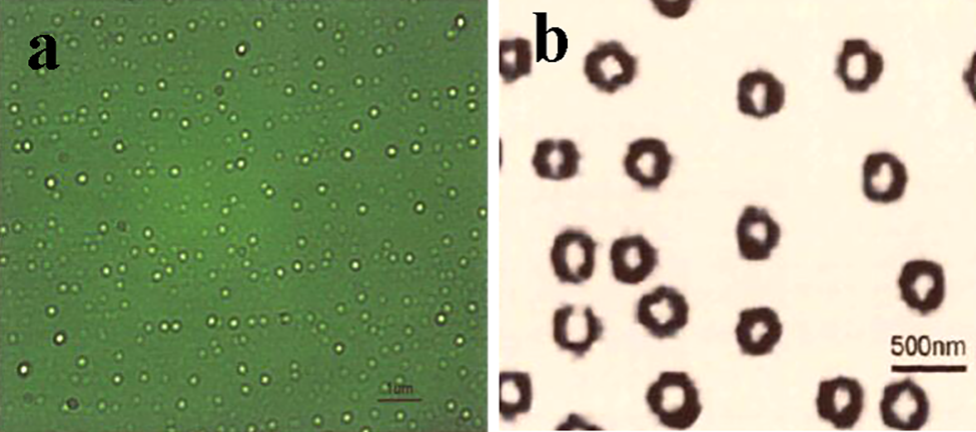
**Morphology of NBs prepared using optimal conditions under a scanning electron microscope (a, ×10000) and fluorescence microscope (b, ×100)**.

We identified the following optimum conditions for preparing NBs using uniform design and multiple regression analyses: a high shear rate of 3000 rpm, high shear time of 8 min, DPPC concentration of 20 mg/50 ml, and T_80_/S_60_ of 1. After optimizing the preparation conditions, the particle size of NBs were found to range from 208 to 416 nm, with an average of 335±5 nm. Moreover, based on fluorescence or scanning electron microscope observation, the particles were approximately uniform in size, complete in morphology and did not accumulate (Fig 1).

### Effects of NBs on cell growth

**Fig 2:**
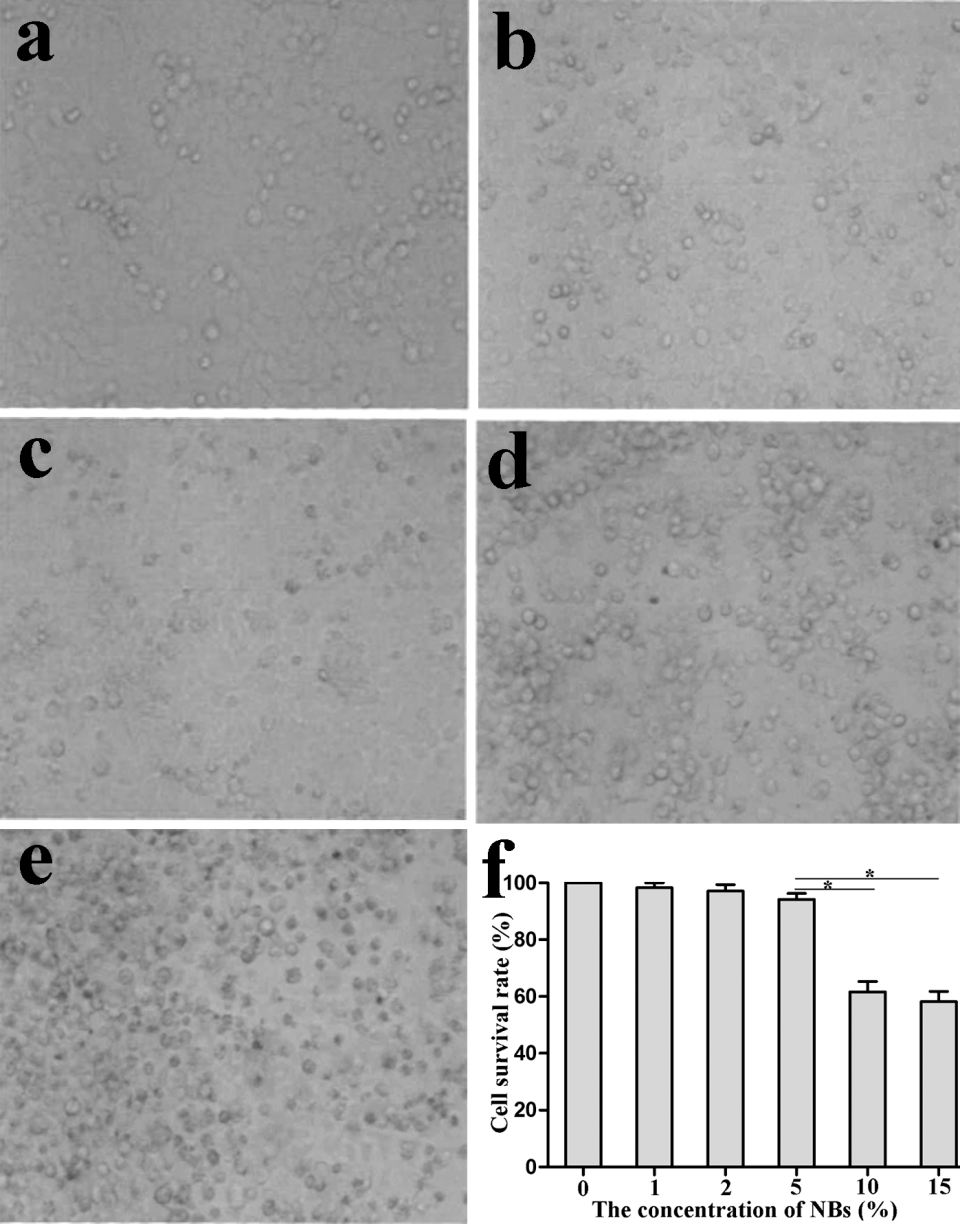
HepG2 cell growth in the presence of different concentrations of NBs (×100). a. HepG2 cells without NBs, b. HepG2 cells with 2% NBs, c.HepG2 cells with 5% NBs, d. HepG2 cells with 10% NBs, e. HepG2 cells with 15% NBs, f. MTT assay detection of the cytotoxic effect of NBs. The cells presented better growth behavior in the presence of ≤5% NBs (*P<0.05).

The growth of HepG2 cells was observed under an inverted microscope after 24 h of culture with different concentrations of NBs. The cells exhibited better growth behavior at concentrations less than or equal to 5%. However, when the concentration was greater than 5%, cell growth was significantly altered, with an obvious increase in the level of intracellular particulate matter (Fig 2, some data not shown). Moreover, the cell survival rate was also confirmed using the MTT assay (*P*<0.05, Fig 2F). Thus, the concentration of NBs used in subsequent experiments was 5%.

### Differences in GFP expression in cells transfected with NBs and liposomes

**Fig 3:**
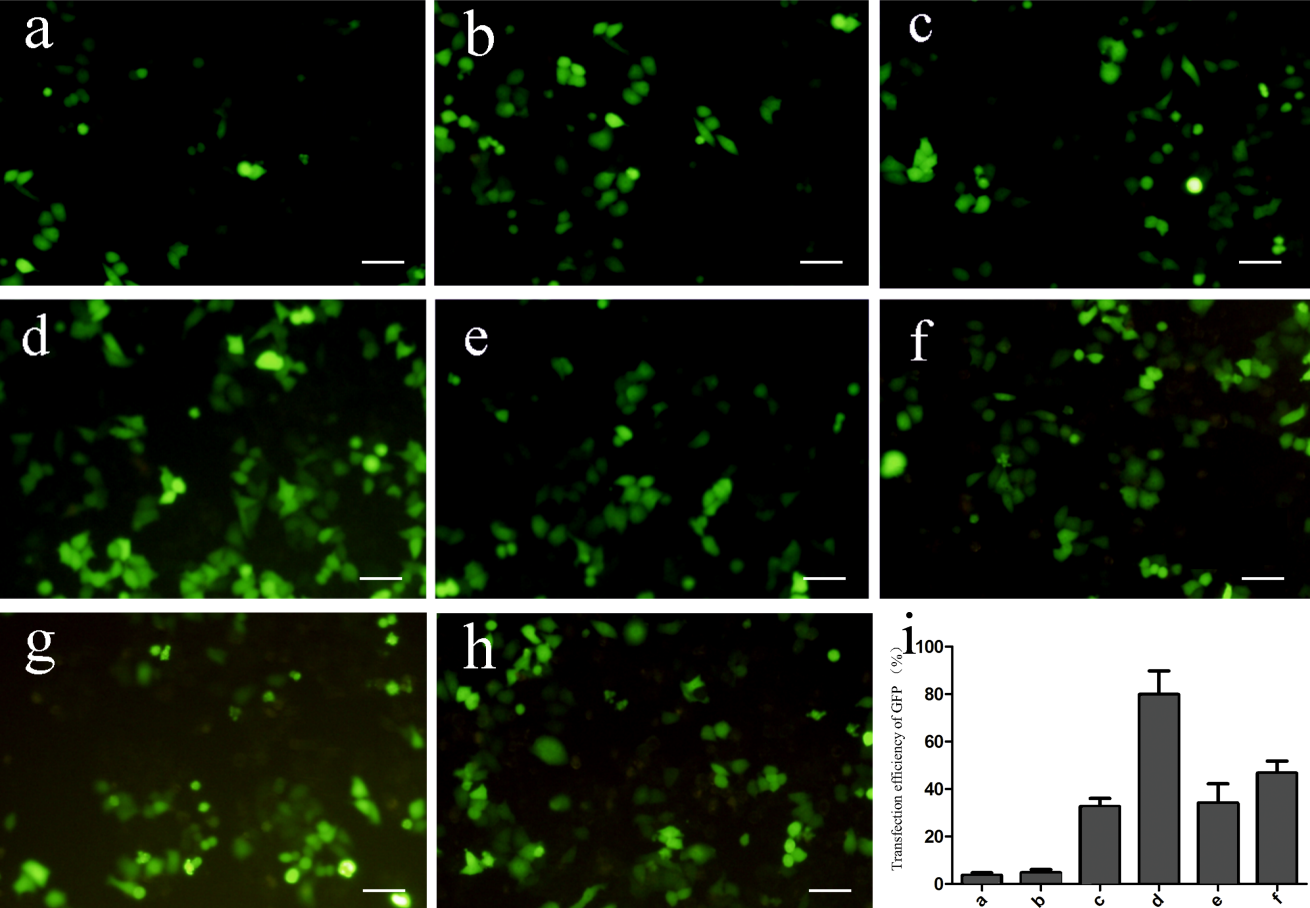
Differences in GFP transfection efficiencies of NBs and liposomes (×200) and GFP transfection with or without ultrasound irradiation (×200). a. 8 μg of plasmid; b. 8 μg of plasmid and ultrasound irradiation; c. NBs and 8 μg of plasmid; d. NBs, 8 μg of plasmid and ultrasound irradiation; e. liposomes and 8 μg of plasmid; f. liposomes, 8 μg of plasmid and ultrasound irradiation; g. NBs and 4 μg of plasmid; h. liposomes and 4 μg of plasmid; i. FCM assessment of GFP expression in transfected cells with or without ultrasound irradiation; expression in group d is significantly higher than in other groups (*P<0.05).

Different concentrations of plasmid were mixed with 5% NBs or liposomes and then transfected into HepG2 cells. Using fluorescence microscopy, more GFP-positive cells were observed in the groups transfected with NBs/liposomes (Groups 2, 3, 4, and 5) than in those transfected with the plasmid alone (Group 1). However, no differences were observed between Groups 2 and 3 and Groups 4 and 5 or among Groups 2, 3, 4, and 5 (Fig 3A, 3C, 3E, 3G and 3H). These results were verified by FCM (transfection efficiency, means±SEM, Table 2) and RT-PCR (2^-ΔΔct^, Table 2).

**Table 2:** Differences in GFP expression between cells transfected with NBs and liposomes.

Group	1	2	3	4	5
Efficiency (%)	3.61±1.11	32.61±3.42*	37.24±3.58*	34.12±3.58*	36.70±9.12*
2^-ΔΔct^	0	2.41*	2.56*	2.20*	2.50*

*P<0.05, compared to Group 1.

### GFP plasmid transfection efficiency with or without ultrasound irradiation

We also observed more GFP-positive cells in Group d (ultrasonic NB-mediated transfection) than in the groups not treated with NBs and ultrasound irradiation (*P*<0.05, Fig 3A, 3B, 3C, 3D, 3E and 3F). FCM analyses confirmed the fluorescence microscopy data (Fig 3I).

### Apoptosis-inducing and “bystander” effects of the PNP/fludarabine suicide gene system

**Fig 4:**
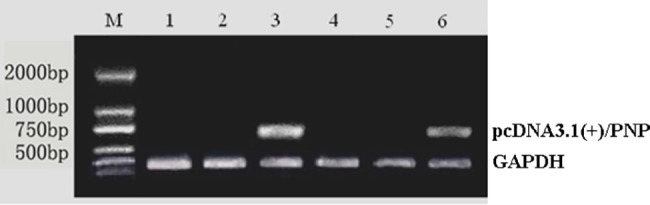
RT-qPCR detection of PNP expression in different groups. M. marker. 1. HepG2 cells; 2. HepG2-pcDNA3.1(+) cells; 3. HepG2-pcDNA3.1(+)/PNP cells; 4. SMMC7721 cells; 5. SMMCC7721-pcDNA3.1(+) cells; 6. SMMCC7721-pcDNA3.1(+)/PNP cells. Band 3 and 6 show the recombinant pcDNA3.1(+)/PNP plasmid was successfully transferred into HCC cells.

In our study, the results of recombinant plasmid PCR showed a specific band at approximately 750 bp, which corresponded to the amplified PNP band (data not shown). Thus, PNP was successfully cloned into the pcDNA3.1(+) plasmid. The same method was also used to demonstrate that the recombinant pcDNA3.1(+)/PNP plasmid was successfully transferred into HCC cells and that the PNP gene was expressed in G418-resistant cells (Fig 4).

**Fig 5:**
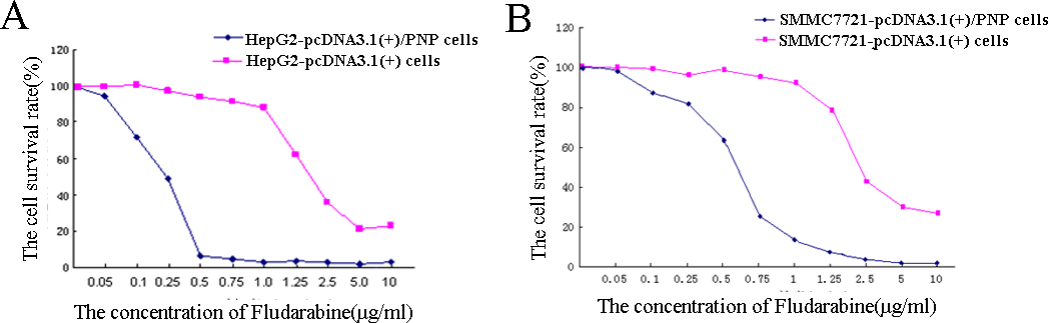
MTT bioassay detection of the cellular growth inhibition effect of the PNP/fludarabine suicide gene system.

Based on the results of MTT assays, the PNP/fludarabine suicide gene system exerted notable cytotoxicity toward HepG2 and SMMC7721 cells, particularly the former (Fig 5). The relative survival rates of HepG2+fludarabine, HepG2-pcDNA3.1/PNP+fludarabine, SMMC7721+fludarabine, and SMMC7721-pcDNA3.1/PNP+fludarabine cells were high at fludarabine concentrations of 0.05–0.2 μg/ml; that is, fludarabine had little effect on cell growth. At a fludarabine concentration ≥0.5 μg/ml, pcDNA3.1/PNP-transfected HepG2 cells presented a lower survival rate (<10%) than pcDNA3.1-transfected cells (*P*<0.05). At ≥0.75 μg/ml fludarabine, pcDNA3.1/PNP-transfected SMMC7721 cells also presented a lower survival rate than pcDNA3.1-transfected cells (*P*<0.05). Moreover, the proliferation of cells in these four groups was significantly restricted at ≥2.5 μg/ml fludarabine (*P*<0.05). Thus, 0.75 μg/ml fludarabine used in the subsequent experiment to detect apoptosis.

After treatment with 0.75 μg/ml fludarabine, the apoptosis rates of the blank control (HepG2/SMMC7721 cells) and HepG2/SMMC7721-pcDNA3.1 cells were substantially lower than those of HepG2/SMMC7721-pcDNA3.1/PNP cells (Table 3). Based on these results, the PNP/fludarabine suicide gene system induces marked HCC apoptosis in HepG2/SMMC7721 cells, which would inhibit tumor growth.

**Table 3:** Apoptosis rates of HCC cells treated with fludarabine (%).

Cells	Blank control	pcDNA3.1	pcDNA3.1/PNP
HepG2	1.24	6.47	41.34
SMMC7721	7.21	10.25	53.15

**Fig 6:**
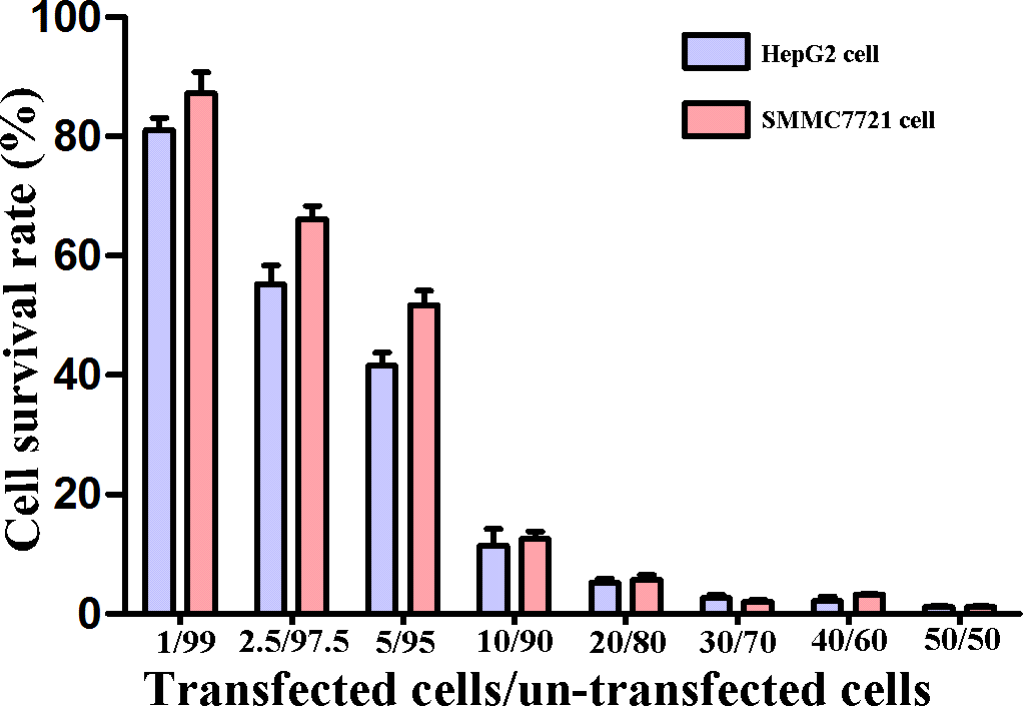
The “bystander” effect of the PNP/fludarabine suicide gene system. The cell survival rate decreased with the increasing ratio of transfected/non-transfected cells (*P*<0.05).

The “bystander” effect is a unique, distinct advantage of suicide gene systems and distinguishes them from other gene therapies. In our study, the MTT assay was performed on cells treated with 0.75 μg/ml fludarabine to detect this effect. At a ratio of transfected to non-transfected HCC cells of 5/95, the cell survival rate was only 40–50%, and the cell survival rate was less than 20% when the ratio was 10/90. These illustrated that the cell survival rate decreased with the increasing ratio of transfected/non-transfected cells (The trend *χ*^2^ is 10024.93(HepG2), 8627.61(SMMC7721); *P* = 0.000, Fig 6). Thus, the PNP/fludarabine suicide gene system exerts an obvious bystander effect to inhibit HepG2/SMMC7721 cell growth.

## Discussion

In this study, we sought to uncover an optimal method for producing ultrasonic NBs and to determine whether the ultrasonic NB-mediated PNP/fludarabine suicide gene system is an effective novel approach for the treatment of HCC. Indeed, the findings offer a new NB-mediated method for HCC gene therapy, particularly because of the bystander effect of PNP/fludarabine.

MBs (usually 2–4.5 μm) have been used as promising ultrasound contrast agents and drug/gene carriers for ultrasonic imaging and drug/gene delivery, respectively. However, the tumor neovasculature is an imperfect structure, with a maximum diameter of approximately only 380–780 nm[13], thus, limiting the application of some MBs due to their large diameters. In contrast, NBs with diameters between 208 and 416 nm, such as those used in our study, would more easily escape from the vasculature through leaky endothelial tumor tissue and then accumulate in certain solid tumors. S_60_ and T_80_ are nonionic surfactants that stabilize the NB membrane and reduce surface tension. A composite membrane formed by a mixture of phospholipids not only substantially increases the NB stability[14] but also promotes biocompatibility and opportunities for surface functionalization, both of which are advantageous for specific targeting to tissues and for modulating mechanical responses to localized ultrasound-induced destruction[15]. These findings provide a good foundation for subsequent experiments.

We found higher levels of GFP expression in cells transfected with an NB/liposome-mediated plasmid than in cells transfected with the plasmid alone; ultrasound irradiation improved GFP transfection efficiency in HepG2 cells, and the effect was more apparent when ultrasound irradiation was combined with NBs. No significant differences in GFP transfection efficiency were observed between NBs and liposomes in the absence of ultrasound irradiation or between 4 μg and 8 μg of plasmid with NBs/liposomes. These results demonstrate the effectiveness and security of gene transfer via NBs and their superiority compared to traditional vectors. The use of traditional gene-transfer vectors, such as viral vectors, is restricted due to immunogenicity, tumorigenicity, or amplicon infection[16, 17]. Accordingly, the use of MB/NB ultrasound agents as drug/gene carriers has received increasing attention[18, 19], not only because they are secure and efficient but also because they are specifically distributed to irradiated tissues or target tissues for fixed-point release by ultrasound irradiation[20] or carrying specific targeted markers[21], especially NBs with smaller diameter. In this study, 5% NBs were not cytotoxic toward HepG2 cells, and plasmid DNA combined with 5% NBs was more efficiently transfected. Intravenously injected MBs were previously shown to accumulate and remain in the target tissue for long periods after passing through the circulation[22]. Although the gene transfection mechanism mediated by ultrasound microbubbles remains unclear, ultrasonic acoustic cavitation and sonoporation are likely the fundamental mechanisms involved[23, 24]. Ultrasonic mechanical waves are believed to alter cell membrane permeability or generate transient pores on the cell membrane and might thus increase uptake of macromolecules such as plasmid DNA[25]. In addition, MBs/NBs are destroyed by ultrasound irradiation and release their cargo, i.e., a drug or gene, which may be applied for targeted drug/gene delivery to reduce undesirable side effects[20].

HCC is a frequently occurring malignant tumor with an increasing incidence due to chronic HBV/HCV infection, exposure to aflatoxins, and cirrhosis[26]. Despite the variety of treatment options, including surgical resection, ablation, chemotherapy, liver transplantation, or transcatheter arterial chemoembolization or embolization, the prognosis of patients with HCC is poor due to the low efficacy of treatment or drug resistance[27]. Nonetheless, targeted gene therapy, such as the ultrasonic NB-mediated PNP/fludarabine suicide gene system, may offer a novel strategy for HCC therapy. *E*. *coli* PNP converts purine or adenosine analogs such as fludarabine into freely diffusible metabolites, which are highly toxic to both dividing and non-dividing cells, and might thus be beneficial for solid tumors with a small number of proliferating cells. As shown in a previous study, 0.5–1 μg/ml fludarabine killed 100% of PNP-expressing cells, with no detectable toxicity toward control cells, and expression of PNP in as few as 10% of HCC cells induced the efficient killing of most bystander cells because PNP metabolites are able to diffuse across cell membranes[28].

In our study, we used PNP/fludarabine as a suicide gene system and assessed gene transfer with the aid of ultrasonic NBs. At ≥0.5 μg/ml fludarabine, pcDNA3.1/PNP-HepG2 cells presented a lower survival rate than pcDNA3.1-HepG2 cells, and SMMC7721 cells presented the same trend at ≥0.75 μg/ml fludarabine. The apoptosis rates of HepG2/SMMC7721-pcDNA3.1/PNP cells were also substantially increased. Based on these findings, the PNP/fludarabine suicide gene system is highly cytotoxic toward HCC cells, particularly HepG2 cells, which express high levels of α-fetal protein (AFP)[29], and only in the presence of low fludarabine concentrations. All those may guide the clinical treatment of HCC. Moreover, a 10–20% decrease in cell survival was observed when only 5–10% of transfected cells were PNP positive. This mechanism is called the “bystander effect”, which appears to be related to cell death in fludarabine-treated pcDNA3.1/PNP-positive cells. The potent cytotoxic activity of PNP upon administration of the prodrug fludarabine and its strong bystander effects on adjacent cells might potentiate tumor destruction and inhibit tumor proliferation. According to previous studies, the herpes simplex virus thymidine kinase (HSV-TK)/ganciclovir (GCV) suicide gene system has little efficacy in clinical practice, mostly due to the low targeting efficiency, the absence of the “bystander effect” and poor gene-transfer efficiency in tumors[5]. Thus, the “bystander effect” appears to be essential for the success of suicide gene therapy. Although cell-to-cell contact was found to be required for the TK/GCV-induced “bystander effect” because of the inability of phosphorylated GCV to diffuse through lipid membranes[30], tumors with cystic and necrotic areas may limit this efficiency. The potential mechanisms responsible for the “bystander effect” are as follows. 1). Apoptosis: Apoptotic bodies generated by the prodrug can be endocytosed by adjacent cells, leading to a subsequent cytotoxic effect. 2). Gap junctions: The mechanism underlying the “bystander effect” of the HSV-TK/GCV suicide gene system was attributed to gap junctions, but the toxic product of PNP is a non-phosphorylated purine base, which can diffuse through the membrane freely without the need for cell or gap junctions. 3. Human immunity: The human immune system is required to kill tumor cells.

Our current study has several limitations. For example, rather than evaluating different intensities, times, and methods, we adopted the safest intensity and duration of ultrasonic irradiation according to ultrasonography. Because HCC is studied most in our lab, the tumor cell lines of this study are too unitary, and we should validate the findings at different cancer cell lines or fludarabine resistant cell lines to increase persuasion, especially at multiple HCC lines. We also should compare it with other nano-particle and liposomes to increase the rigor of this study. Most important, this study is only for in vitro experiments, and the results must be further confirmed in animal models before the system can be applied in clinical practice. We have made nude mouse models of orthotopic and subcutaneous HCC(Supporting Information), and we will perform animal experiments in a follow-up study.

In summary, we reveal the feasibility of applying the ultrasonic NB-mediated PNP/fludarabine suicide gene system as a new approach for treating HCC in vitro. NBs not only facilitate excellent contrast enhancement for diagnosis but also have stronger penetration ability than the currently used MBs, allowing them to extravasate from tumor vessels and thus facilitating targeted ultrasound imaging and therapy. The killing effect and “bystander effect” of the PNP/fludarabine suicide gene system make it superior to other systems, such as HSV-TK/GCV. Efficient optimization of the expression of the therapeutic gene is possible using this strategy, resulting in improved and potentially enhanced HCC treatment.

## Supporting information

S1 Table HepG2 cell growth in the presence of different concentrations of NBs. (DOCX)

S2 Table GFP plasmid transfection efficiency with or without ultrasound irradiation was detected by FCM. (DOCX)

S3 Table Apoptosis rates of HCC cells treated with fludarabine were detected by FCM (%). (DOCX)

S4 Table The “bystander” effect of the PNP/fludarabine suicide gene system. (DOCX)

S1 Fig HCC animal models has been made.(A: subcutaneous HCC of nude mouse; B:orthotopic HCC of nude mouse).
(DOCX)
