# Supplementary material for: An ultrasonic nanobubble-mediated PNP/fludarabine suicide gene system: A new approach for the treatment of hepatocellular carcinoma
Source: PLoS One. 2018 May 2;13(5):e0196686. doi: 10.1371/journal.pone.0196686 (PMC5931662; doi:10.1371/journal.pone.0196686)
Supplement: S1 Table — (DOCX) [file pone.0196686.s001.docx]

| The concentration of NBs(%) | Cell survival rate(%)  n=1000 | | |
| --- | --- | --- | --- |
| 0 | 100 | 100 | 100 |
| 1 | 98.3 | 96.6 | 100 |
| 2 | 94.8 | 99.4 | 97.1 |
| 5 | 92.1 | 96.3 | 94.2 |
| 10 | 61.6 | 65.3 | 57.9 |
| 15 | 61.8 | 58.2 | 54.6 |

**S1** **Table. HepG2 cell growth in the presence of different concentrations of NBs**
