# Supplementary material for: An ultrasonic nanobubble-mediated PNP/fludarabine suicide gene system: A new approach for the treatment of hepatocellular carcinoma
Source: PLoS One. 2018 May 2;13(5):e0196686. doi: 10.1371/journal.pone.0196686 (PMC5931662; doi:10.1371/journal.pone.0196686)
Supplement: S2 Table — (DOCX) [file pone.0196686.s002.docx]

**S2 Table. GFP plasmid transfection efficiency with or without ultrasound irradiation was detected by FCM**

| Group | a | b | c | | d | e | f |
| --- | --- | --- | --- | --- | --- | --- | --- |
| Efficiency (%) | 3.61±1.11 | 4.74±1.32^*^ | 32.61±3.42 | 79.9±9.93 | | 34.12±8.06 | 46.7±5.12 |

**
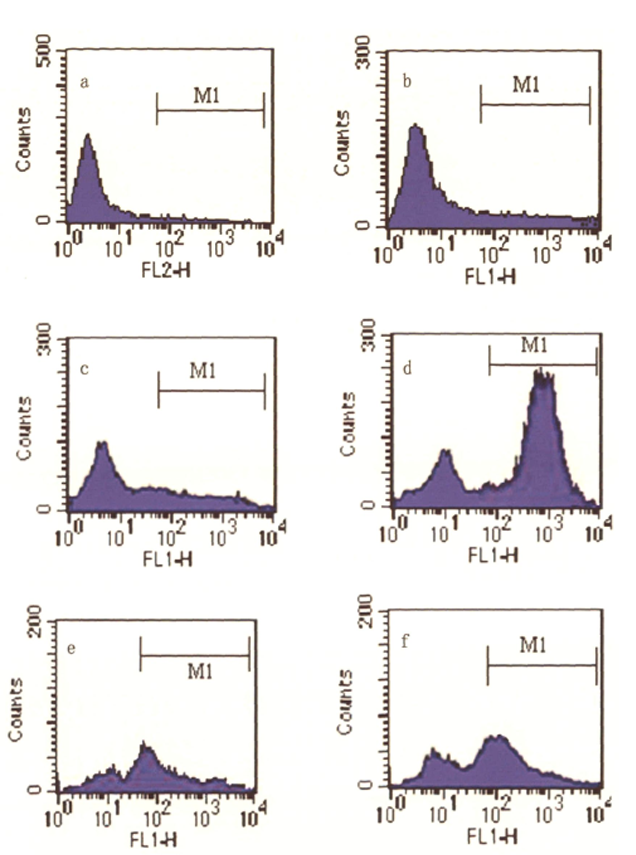
**
