# Supplementary material for: An ultrasonic nanobubble-mediated PNP/fludarabine suicide gene system: A new approach for the treatment of hepatocellular carcinoma
Source: PLoS One. 2018 May 2;13(5):e0196686. doi: 10.1371/journal.pone.0196686 (PMC5931662; doi:10.1371/journal.pone.0196686)
Supplement: S3 Table — (DOCX) [file pone.0196686.s003.docx]

**S3 Table. Apoptosis rates of HCC cells treated with fludarabine were detected by FCM(%)**

| Cells | Blank control | pcDNA3.1 | pcDNA3.1/PNP |
| --- | --- | --- | --- |
| HepG2 | 1.24 | 6.47 | 41.34 |
| SMMC7721 | 7.21 | 10.25 | 53.15 |

**
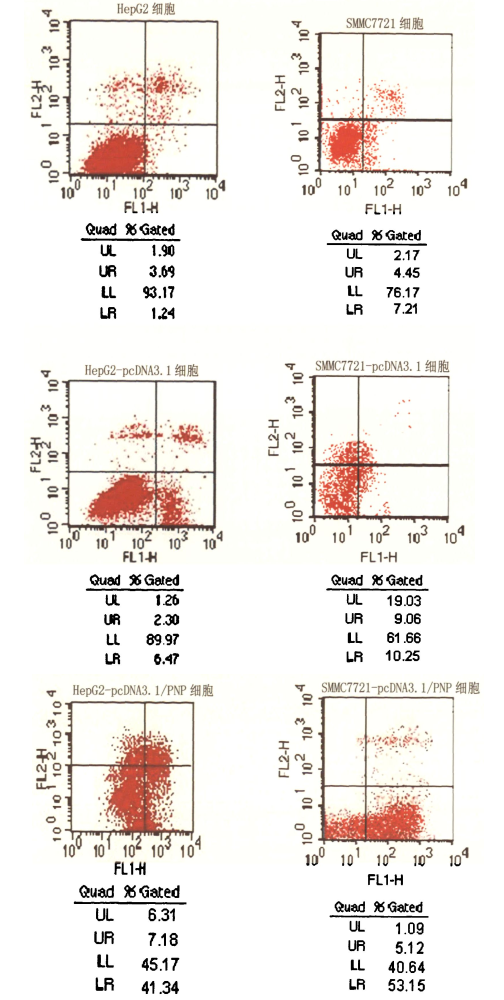
**
