# Supplementary material for: An ultrasonic nanobubble-mediated PNP/fludarabine suicide gene system: A new approach for the treatment of hepatocellular carcinoma
Source: PLoS One. 2018 May 2;13(5):e0196686. doi: 10.1371/journal.pone.0196686 (PMC5931662; doi:10.1371/journal.pone.0196686)
Supplement: S4 Table — (DOCX) [file pone.0196686.s004.docx]

**S4 Table. The “bystander” effect of the PNP/fludarabine suicide gene system.**

| Different cell rate  (Transfected/un-transfected cells) | Cell survival rate(%)  n=3000 | |
| --- | --- | --- |
|  | HePG2 | SMMC7721 |
| 1/99 | 81±2.1 | 87.2±3.6 |
| 2.5/97.5 | 55.2±3.2 | 66.1±2.3 |
| 5/95 | 41.5±2.3 | 51.7±2.4 |
| 10/90 | 11.4±2.8 | 12.5±1.3 |
| 20/80 | 5.2±0.7 | 5.7±0.8 |
| 30/70 | 2.6±0.6 | 2.6±0.4 |
| 40/60 | 2.1±0.8 | 3.2±0.2 |
| 50/50 | 1.1±0.3 | 1.1±0.2 |
