# Supplementary material for: An ultrasonic nanobubble-mediated PNP/fludarabine suicide gene system: A new approach for the treatment of hepatocellular carcinoma
Source: PLoS One. 2018 May 2;13(5):e0196686. doi: 10.1371/journal.pone.0196686 (PMC5931662; doi:10.1371/journal.pone.0196686)
Supplement: S1 Fig — HCC animal models has been made.(A: subcutaneous HCC of nude mouse; B:orthotopic HCC of nude mouse). (DOCX) [file pone.0196686.s005.docx]

**S1 Fig. HCC animal models has been made.(A: subcutaneous HCC of nude mouse; B:orthotopic HCC of nude mouse)**

**
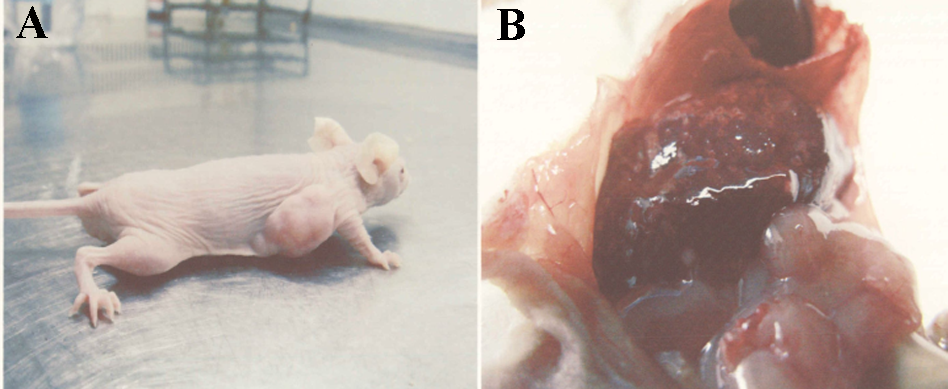
**
